# Long-term benefits of heart rate variability biofeedback training in older adults with different levels of social interaction: a pilot study

**DOI:** 10.1038/s41598-022-22303-z

**Published:** 2022-11-05

**Authors:** Perciliany Martins de Souza, Miriam de Cássia Souza, Luiza Araújo Diniz, Cássia Regina Vieira Araújo, Mariana Lopez, Eliane Volchan, Orlando Fernandes, Tiago Arruda Sanchez, Gabriela Guerra Leal Souza

**Affiliations:** 1 Laboratory of Psychophysiology, Department of Biological Sciences, Institute of Exact and Biological Sciences, Federal University of Ouro Preto, Ouro Preto, Brazil; 2 Graduate Program in Psychology, Federal University of Santa Catarina, Santa Catarina, Brazil; 3 Laboratory of Neurobiology, Carlos Chagas Filho Biophysics Institute, Federal University of Rio de Janeiro, Rio de Janeiro, Brazil; 4 Laboratory of Neuroimaging and Psychophysiology, Department of Radiology, Federal University of Rio de Janeiro, Rio de Janeiro, Brazil

**Keywords:** Neuroscience, Physiology, Psychology, Health care, Risk factors

## Abstract

To test whether heart rate variability (HRV) biofeedback training benefits older adults with different social interaction levels. Methods. 32 older adults (16 were institutionalized and 16 were not). Both groups received 14 sessions, 15 min, 3 times a week, with half of the individuals receiving HRV biofeedback training and the other half receiving control training. The following parameters were assessed immediately before and after training, and 4.5 weeks after the last session (follow-up period): aerobic conditioning, anthropometric data, emotional scores, and HRV components. Results. Before the training, the institutionalized individuals had higher scores of loneliness (p < 0.01) and depression (p < 0.0001) and lower social touches (p < 0.0001), body mass (p = 0.04), and body fat percentage (p = 0.002) than the non-institutionalized individuals. HRV biofeedback improved symptoms of depression in both groups. HRV improved only in the non-institutionalized group, and loneliness only in the institutionalized group. Lastly, all changes persisted after the follow-up period. Conclusions. HRV biofeedback training was effective in improving symptoms of depression in older adults. Improvement of HRV and loneliness was dependent on the level of social interaction.

## Introduction

Biofeedback training is a therapeutic tool that is useful in the teaching and learning of physiological and psychological processes of self-regulation^1^. It is an assisted learning method used to modify physiological functions over which one has little conscious control, such as respiratory rate, heart rate, and cerebral impulse activity^2^. Heart rate variability (HRV) biofeedback is a type of biofeedback that specifically involves respiratory rate modification (decrease in frequency and increase in amplitude) to increase synchronization between respiratory rate and heart rate. This synchronization allows an increase in the amplitude of HRV^3^.

HRV is a non-invasive measure extracted from electrocardiogram recording that reflects the continuous oscillation of the RR intervals as a function of the action of the sympathetic and parasympathetic branches on the sinoatrial node. RR intervals decrease during inspiration and increase during expiration^4,5^. HRV has been proposed as a marker of physical and mental health^6^ and, more specifically, as an important marker of social engagement^7,8^.

The social relations of older adults may change for a variety of reasons, including geographical migration of family members or friends, death or disability among members of their social networks, and reduction of physical or cognitive abilities^9^. Another important factor is institutionalization, which is in general linked to the feeling of social rejection^10–12^. Moreover, loneliness, depression (and other mental disorders), and social rejection are strongly correlated, and the negative impact of these variables on the lives of older adults can be significant^13–15^. Lastly, although mental health variables are negatively correlated with HRV, they may be positively modified through HRV biofeedback, as shown in adults^10,16^.

To our knowledge, there are no studies to date on the impact of HRV biofeedback on the health of the elderly population and, more specifically, on the role of the level of social interaction. Given the above, the objective of this pilot study was to test whether HRV biofeedback training benefits the health of older adults with different levels of social interaction. We specifically investigated whether: (i) there are differences in terms of aerobic conditioning, anthropometric data, emotional scores, and HRV components between institutionalized and non-institutionalized older adults; (ii) HRV biofeedback training improved these parameters among older adults; (iii) these improvements were dependent on institutionalization; and (iv) the benefits persisted after the training intervention.

## Results

The total sample of the study comprised 32 volunteers, of whom 16 were institutionalized and 16 were not. The non-institutionalized group was composed of individuals who were members of a recreation club for older adults for 8.75 ± 8.01 years, in which four were men and 12 were women, with a mean age of 72.50 (69.00/76.00) years. The institutionalized group lived in the institution for 6.87 ± 6.94 years and included nine women and seven men with a mean age of 69.50 (67.50/75.50) years.

An investigation of the characteristics of the two study groups was performed before starting the interventions and significant differences were found between them. The comparisons are shown in Table 1.

**Table 1:** Characteristics of the sample before the intervention.

Variables	Groups		p-value
	Non-institutionalized (N = 16) Mean ± SD/median (P25/P75)	Institutionalized (N = 16) Mean ± SD/median (P25/P75)	
Aerobic conditioning (m)	400.00 (360.00/44.00)	385.00 (310.00/400.00)	0.16
**Anthropometric**			
Height (m)	1.60 (1.57/1.62)	1.59 (1.44/1.67)	0.67
Body mass (kg)	68.58 ± 11.68	60.48 ± 9.73	0.04*
BMI (kg/m^2^)	27.08 ± 3.43	24.74 ± 3.45	0.06
Body fat percentage (%)	28.00 (25.00/29.50)	17.00 (16.00/23.00)	0.002*
**Emotional**			
Total touch	39.00 (32.00/45.50)	31.00 (28.00/34.00)	0.0005*
Loneliness	3.00 (2.00/7.00)	23.00 (20.00/29.50)	0.000005*
Depression	4.00 (4.00/4.00)	5.00 (4.50/6.50)	0.0003*
**Physiological**			
RMSSD (ms)	72.60 ± 47.09	67.03 ± 43.39	0.75
SDNN (ms)	69.08 (28.71/94.05)	49.04 (31.14/83.67)	0.57
pNN50 (%)	25.63 (3.26/41.40)	30.81 (4.54/53.66)	0.92
SD1 (ms)	51.40 ± 33.34	47.46 ± 30.72	0.75
HF (ms^2^)	1668.40 (155.34/2145.95)	832.76 (167.59/2383.20)	0.87

*N* number of individuals, *SD* standard deviation, *P25* 25th percentile, *P75* 75th percentile, *Kg* kilogram, *BMI* body mass index, *cm* centimeters, *m* meters, *kg/m*^*2*^ kilogram per square meter, *RMSSD* root mean square of the successive differences between the RR intervals, *SDNN* standard deviation of all RR intervals, *pNN50* percentage of successive differences between the RR intervals that are > 50 ms, *SD1* standard deviation 1, *ms* milliseconds.

*Significant difference between the groups (*p < .05).

The institutionalized and non-institutionalized groups differed in assessment 1 with indications of being different groups before the start of any intervention. Therefore, it was decided that all subsequent analyses should be conducted separately for each group (institutionalized and non-institutionalized).

After this characterization, we sought to determine whether training with biofeedback was able to promote changes in the variables evaluated when compared to the control group, and, if so, whether these changes persisted after a follow-up period. The data collected before (assessment 1) and after the 14 training sessions (assessment 2) and after the follow-up period (assessment 3) were compared between the biofeedback group and the control group within the non-institutionalized sample and then within the institutionalized sample.

### Non-institutionalized group

#### Anthropometric and aerobic conditioning variables

In the non-institutionalized sample, the ANOVAs of the anthropometric and aerobic conditioning variables showed an effect of time (assessments 1, 2, and 3) on body fat percentage (F(2, 28) = 5.18, p = 0.01, *d* = 0.27) but not on body mass (F(2, 28) = 2.31, p = 0.11, *d* = 0.14), BMI (F(2, 28) = 2.39, p = 0.10, *d* = 0.15), and aerobic test (F(2, 28) = 0.92, p = 0.40, *d* = 0.06). There was a reduction in body fat percentage in the second and third assessments relative to the first assessment. There was an effect of group (control and biofeedback) on body mass (F(1, 14) = 4.62, p = 0.04, *d* = 0.25) biofeedback group with higher body mass than the control group but not on BMI (F(1, 14) = 3.57, p = 0.07, *d* = 0.20), aerobic test (F(1, 14) = 0.31, p = 0.58, *d* = 0.02), and body fat percentage (F(1, 14) = 4.29, p = 0.05, *d* = 0.23). There was no effect of the interaction between time and group on body mass (F(2, 28) = 0.33, p = 0.72, *d* = 0.02), BMI (F(2, 28) = 0.26, p = 0.76, *d* = 0.02), aerobic test (F(2, 28) = 0.99, p = 0.38, *d* = 0.07), and body fat percentage (F(2, 28) = 0.12, p = 0.88, *d* = 0.009).

#### Emotional variables

**Figure 1:**
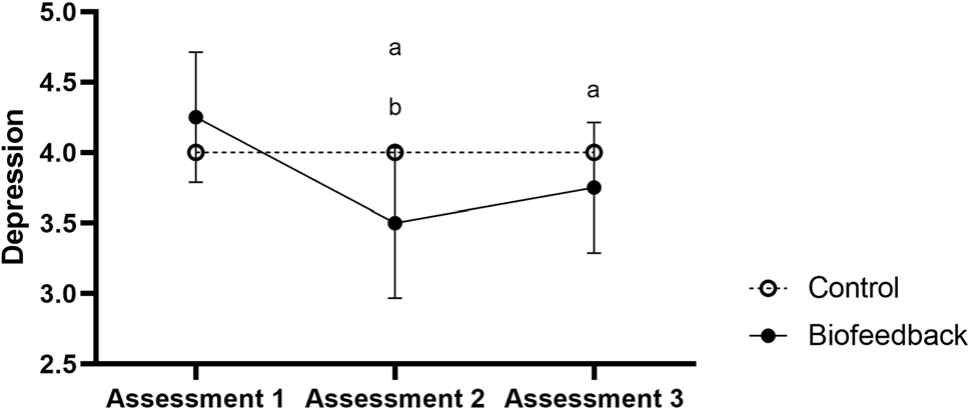
Symptoms of depression during assessments 1, 2, and 3 in the group that received biofeedback training (solid black line) and in the group that received the control training (dotted black line) in the non-institutionalized sample. ^a^Significant difference of assessment 2 and 3 in comparison to assessment 1 (< 0.05) and ^b^significant differences between groups (< 0.05).

No main effects of time (assessments 1, 2, and 3) were observed in the ANOVAs of the emotional variables on loneliness (F(2, 28) = 2.03, p = 0.14, *d* = 0.13) and total touch (F(2, 28) = 1.24, p = 0.30, *d* = 0.08). In addition, there was no effect of group (control and biofeedback) on loneliness (F(1, 14) = 0.40, p = 0.53, *d* = 0.03), total touch (F(1, 14) = 0.86, p = 0.36, *d* = 0.06), and symptoms of depression (F(1, 14) = 1.75, p = 0.20, *d* = 0.11). No interaction between time and group was observed on loneliness (F(2, 28) = 2.03, p = 0.14, *d* = 0.13) and total touch (F(2, 28) = 2.80, p = 0.07, *d* = 0.17). However, there was a main effect of time (F(2, 28) = 7.00, p = 0.003, *d* = 0.33) and of the time and group interaction (F(2, 28) = 7.00, p = 0.003, *d* = 0.33) on symptoms of depression, which indicates a reduction in the symptoms of depression after biofeedback training; moreover, this reduction persisted after the training intervention (follow-up) (Fig. 1).

#### HRV components

**Figure 2:**
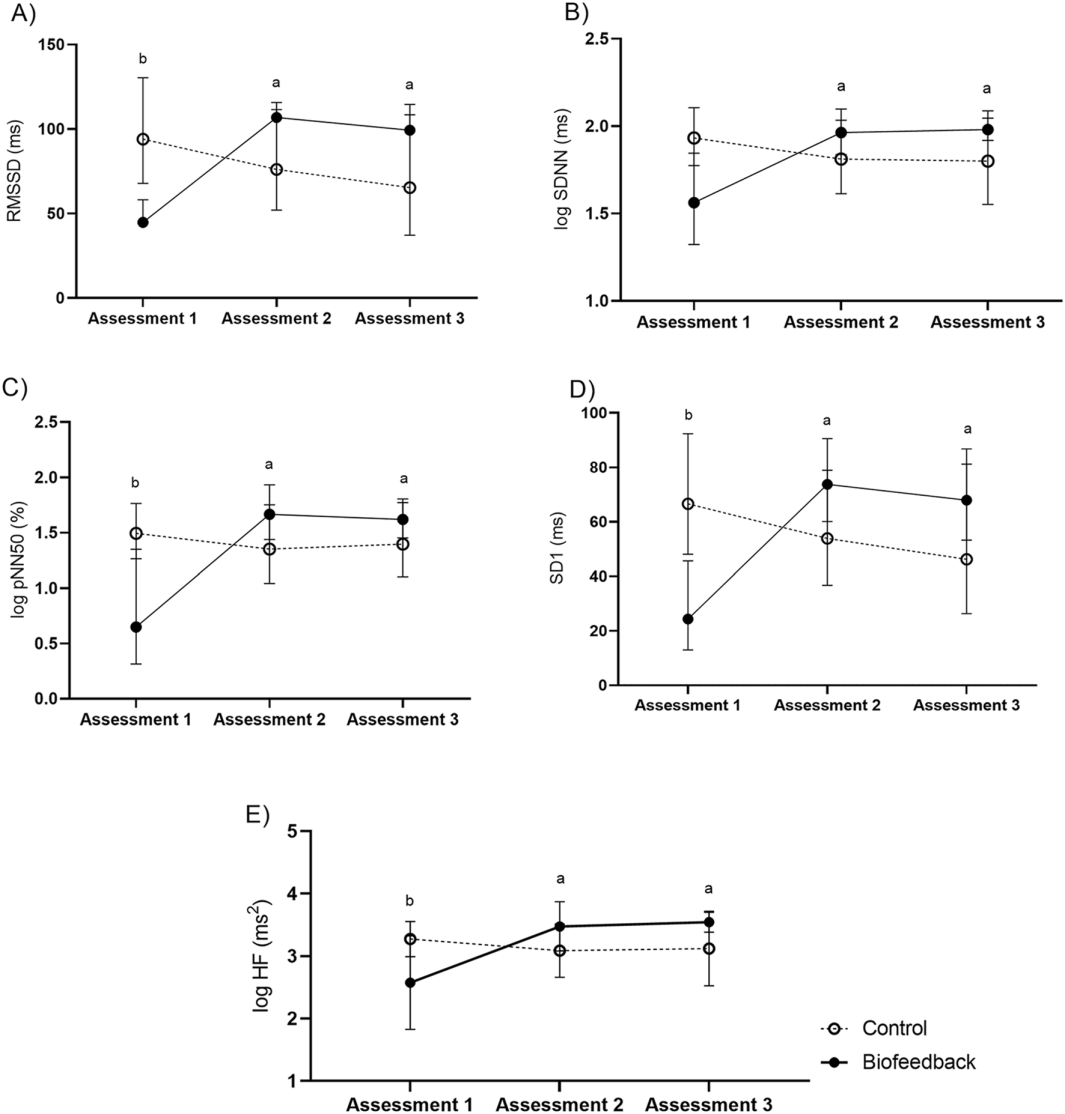
(**A**) Values of the root mean square of the successive differences between the RR intervals (RMSSD) in ms; (**B**) values of logarithmic of the standard deviation of all R-R intervals (log SDNN) in ms; (**C**) values of logarithmic of the percentage of the successive differences between the R-R intervals that are > 50 ms (log pNN50) in ms; (**D**) values of standard deviation 1 (SD1) in ms; (**E**) values of logarithmic of high frequency (log HF) in ms^2^. All these components were evaluated in assessments 1, 2, and 3 in the group that received biofeedback training (solid black line) and in the group that received the control training (black dotted line) in the non-institutionalized sample. ^a^Significant difference of assessment 2 and 3 in comparison to assessment 1 (< 0.05) and ^b^significant differences between groups (< 0.05).

The ANOVAs of HRV showed a main effect of time (assessments 1, 2, and 3) on the RMSSD (F(2, 28) = 3.78, p = 0.03, *d* = 0.21), SDNN (F(2,28) = 3.69, p = 0.03, *d* = 0.20), pNN50 (F(2, 28) = 5.19, p = 0.01, *d* = 0.27), SD1 (F(2, 28) = 3.78, p = 0.03, *d* = 0.21) and HF (F(2,28) = 5.55, p < 0.01, *d* = 0.28) . There was no main effect of group (control and biofeedback) on the variables RMSSD (F(1, 14) = 0.04, p = 0.83, *d* = 0.003), SDNN (F(1, 14) = 0.03, p = 0.86, *d* = 0.002), pNN50 (F(1, 14) = 0.04, p = 0.83, *d* = 0.003), SD1 (F(1, 14) = 0.04, p = 0.83, *d* = 0.003) and HF (F(1, 14) = 0.04, p = 0.84, *d* = 0.003). There was an effect of the interaction between time and group on all variables: RMSSD (F(2, 28) = 14.99, p < 0.0001, *d* = 0.52) (Fig. 2A), SDNN (F(2, 28) = 12.88, p = 0.0001, *d* = 0.47) (Fig. 2B), pNN50 (F(2, 28) = 11.62, p = 0.0002, *d* = 0.45) (Fig. 2C), SD1 (F(2, 28) = 14.99, p < 0.0001, *d* = 0.52) (Fig. 2D), and HF (F(2, 28) = 11.37, p = 0.0002, *d* = 0.45) (Fig. 2E). The post-tests showed an increase in all these variables after biofeedback training. This gain was sustained in the follow-up period. Moreover, at the beginning of the experiment, the RMSSD, SD1 and HF components were higher in the control group than in the biofeedback group, although this difference disappeared after the training.

### Institutionalized group

#### Anthropometric and aerobic conditioning variables

In the institutionalized sample, the ANOVAs of the anthropometric and aerobic conditioning variables showed a main effect of time (assessments 1, 2, and 3) on body fat percentage (F(2, 28) = 3.50, p = 0.04, *d* = 0.20), with a higher body fat percentage in the last assessment, but not on body mass (F(2, 28) = 0.08, p = 0.91, *d* = 0.006), BMI (F(2, 28) = 0.11, p = 0.88, *d* = 0,008) and aerobic test (F(2, 28) = 1.00, p = 0.37, *d* = 0.07). In addition, there was not a main effect of group (control and biofeedback) on body mass (F(1, 14) = 0.01, p = 0.92, *d* = 0.0003), BMI (F(1, 14) = 0.003, p = 0.95), aerobic test (F(1, 14) = 0.03, p = 0.85, *d* = 0.02), and body fat percentage (F(1, 14) = 0.01, p = 0.91, *d* = 0.0008). Moreover, there was no effect of the interaction between time and group on body mass (F(2, 28) = 0.25, p = 0.77, *d* = 0.02), BMI (F(2, 28) = 0.19, p = 0.82, *d* = 0.01), aerobic test (F(2, 28) = 0.06, p = 0.93, *d* = 0.005), and body fat percentage (F(F(2, 28) = 1.16, p = 0.32, *d* = 0.08).

#### Emotional variables

**Figure 3:**
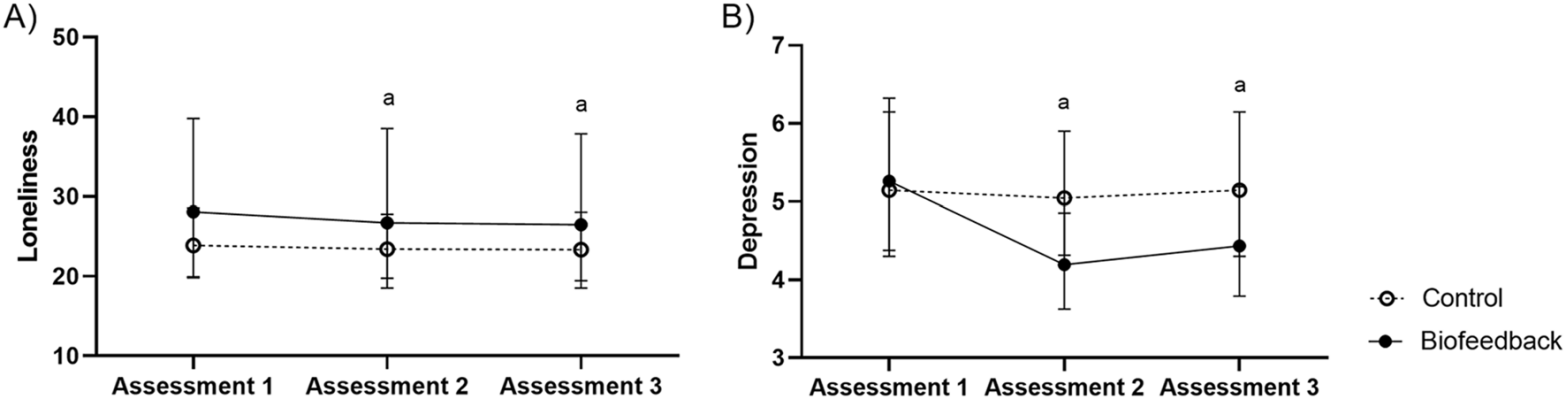
(**A**) Loneliness; (**B**) symptoms of depression. All scales were evaluated in assessments 1, 2, and 3 in the group that received biofeedback training (solid black line) and in the group that received the control training (dotted black line) in the institutionalized sample. ^a^Significant difference of assessment 2 and 3 in comparison to assessment 1 (< 0.05).

With regard to the ANOVAs of the emotional variables, there was an effect of time (assessments 1, 2, and 3) on loneliness (F(2, 28) = 14.77, p < 0.01, *d* = 0.51) and symptoms of depression (F(2, 28) = 10.63, p < 0.01, *d* = 0.43) but not on total touch (F(2, 28) = 1.18, p = 0.32, *d* = 0.07). In addition, there was no effect of group (control and biofeedback) on total touch (F(1, 14) = 0.16, p = 0.69, *d* = 0.01), loneliness (F(1, 14) = 1.05, p = 0.32, *d* = 0.07), or depression (F(1, 14) = 1.07, p = 0.31, *d* = 0.07). There was no effect of the time and group interaction on touch (F(2, 28) = 0.59, p = 0.55, *d* = 0.04). However, there was an effect of the time and group interaction on loneliness (F(2, 28) = 3.76, p = 0.03, *d* = 0.21) and symptoms of depression (F(2, 28) = 7.67, p = 0.002, *d* = 0.35) (Fig. 3). In sum, feelings of loneliness and symptoms of depression decreased after biofeedback training and this reduction persisted after the training ceased.

#### HRV components

The ANOVAs of the variables of the HRV components showed an effect of time (assessments 1, 2, and 3) on RMSSD (F(2, 28) = 8.77, p = 0.001, *d* = 0.39), SDNN (F(2, 28) = 5.28, p = 0.01, *d* = 0.22), and SD1 (F(2, 28) = 8.78, p = 0.001, *d* = 0.39), with the highest values obtained in the second assessment being for all components, but there are not effect of time to pNN50 (F(2, 28) = 2.82, p = 0.07, *d* = 0.16), and HF (F(2, 28) = 2.46, p = 0.10, *d* = 0.15). In addition, there was a main effect of group (control and biofeedback) on RMSSD (F(1, 14) = 1.01, p = 0.32, *d* = 0.07), SDNN (F(1, 14) = 2.36, p = 0.14, *d* = 0.014), pNN50 (F(1, 14) = 1.31, p = 0.27, *d* = 0.07), and SD1 (F(1, 14) = 1.01, p = 0.33, *d* = 0.07), but not to HF (F(1, 14) = 1.10, p = 0.31, *d* = 0.09). Moreover, there was no effect of time and group interaction on the variables RMSSD (F(2, 28) = 0.72, p = 0.49, *d* = 0.05), SDNN (F(2, 28) = 0.33, p = 0.71, *d* = 0.02), pNN50 (F(2, 28) = 0.66, p = 0.52, *d* = 0.04), SD1 (F(2, 28) = 0.72, p = 0.49, *d* = 0.05), and HF (F(2, 28) = 0.31, p = 0.73, *d* = 0.02).

## Discussion

In this pilot study, it was shown that (i) institutionalized older adults had higher baseline scores (before any intervention) regarding symptoms of depression and perceived social isolation and lower scores of social touches, lower body mass, and lower body fat percentage than the non-institutionalized older adults, with no differences in the HRV variables; (ii) HRV biofeedback training improved the symptoms of depression as well as of all HRV components among non-institutionalized older adults, whereas there was an improvement only in perceived social isolation and symptoms of depression in the institutionalized group; (iii) parameters that improved with HRV biofeedback were not all the same in the non-institutionalized and institutionalized groups; (iv) all parameters that were modified by biofeedback training persisted in the follow-up period in both groups.

Peplau^12^ described loneliness as a painful warning sign that the individual’s social relations are deficient. Accordingly, Kross et al.^11^ showed through magnetic resonance imaging that social rejection “hurts” not only metaphorically but also physically because social rejection and physical pain activate the same regions of the brain. Depression has been pertinently associated with loneliness^13,17–20^. These authors reported that the feeling of loneliness is strongly correlated with depression. The stronger the feeling of loneliness, the higher the depression indices or the probability of experiencing some degree of depression^19,20^. Lastly, a review conducted by Mushtaq et al.^21^ reinforced the conclusion that “loneliness can lead to various psychiatric disorders and physical conditions”. We speculate that the findings of Kross et al.^11^ may apply to older adults rejected by their families or by society. Rejection is one of the major causes of institutionalization^14,15^. Thus, the results of these studies are in line with our findings of lower scores of social touches and higher scores of perceived social isolation and depression in the institutionalized group than in the non-institutionalized group, whose social networks were preserved.

HRV biofeedback training improved all HRV components among the non-institutionalized but not among the institutionalized older adults. Several studies with samples of young adults showed that HRV biofeedback training improved HRV parameters^16^. However, there are no studies on the topic with older adults. No study that considered social interaction as a factor that influences the gains generated by biofeedback HRV training was found, regardless of the sampled population. The absence of social interaction in the everyday life of the institutionalized older adults probably prevented them from benefiting more from biofeedback training. Given this low social engagement, the polyvagal theory can provide a consistent theoretical basis for the interpretation of these results^7,22,23^, because only the group with high levels of social interaction (non-institutionalized group) had the vagal components of HRV improved. It is worth noting that these individuals were members of a club that offered activities such as theater, dance, and physical exercise and that the majority also had close partners. These are important factors for physiological correlates of social interaction, including oxytocin, endorphins, and vagal tone^20,24,25^.

HRV biofeedback training improved symptoms of depression in both groups, i.e., it was independent of the level of social interaction. Recent studies such as the meta-analysis by Lehrer et al.^16^ describe the effects of HRV biofeedback training at the central level, in which significant increase in blood flow fluctuations in all areas involved in emotional processing and modulation during training, specifically the limbic system and the cingulate and prefrontal cortices, could explain the improvement of depression-related states. A meta-analysis conducted by Lehrer et al.^16^ included several studies showing that HRV and depression improved after HRV biofeedback training. However, the literature is very scarce in studies with older adults. To our knowledge, there are only two studies in which HRV biofeedback training was used in the older adults in which mental health was evaluated^26,27^. Jester, Rozek and McKelley^26^ performed a 30-min training twice a week for 3 weeks to reduce psychiatric symptoms and improve cognitive functioning. The authors concluded that older adults may benefit from HRV biofeedback training the same way as the younger population. In addition, depression, anxiety, and attentional skills improved significantly after the intervention. This result is very similar to that obtained by Zauszniewski and Musil^27^, who observed a reduction in the levels of stress and depression among elderly women after 4 weeks of biofeedback training.

Another crucial result of this study was that of the follow-up assessment. We aimed to investigate whether the gains obtained from HRV biofeedback training would persist 4.5 weeks after the end of the intervention. Our findings showed that all gains generated by the training persisted after the intervention ended, in both groups (institutionalized and non-institutionalized). A recent meta-analysis confirmed our results, showing that HRV biofeedback training for a few weeks was effective in improving symptoms of depression in several psychophysiological conditions^28^. In one study involving patients with cancer, HRV biofeedback training was used with four to six sessions per week for a variable period, depending on the time required by each individual to reach cardiorespiratory coherence. The authors reported a significant improvement of stress, fatigue, and symptoms of post-traumatic stress disorder and depression relative to a control group, as well as in the maintenance of the cardiorespiratory coherence after 1 week of follow-up^29^. We emphasize that this is also a key and novel point of the study given the scarcity of studies investigating the persistence of the effects of biofeedback training after the follow-up period.

The study had the following limitations: a small sample size, imbalance between the number of men and women, and possible selection bias. To minimize the limitations of the study, (i) each volunteer performed the assessments and training on the same schedule, (ii) the intervention alternated between one individual of the control group and another of the biofeedback group, (iii) selection criteria were lack of volunteers with psychiatric illnesses, severe respiratory or cardiovascular diseases, cognitive impairments, walking difficulties, and smokers, (iv) the researchers who had contact with the volunteers were duly trained and were the same throughout the three assessments, and (v) on the assessment days, the participants were recommended not to ingest caffeine in the 2 h before the intervention and not to perform intense physical exercise and consume alcohol in the 24 h before the intervention.

## Methods

### Sample

The pilot study included 32 older adults of both genders, aged between 63 and 79 years. They were living in a long-term care institution for at least 6 months (institutionalized group: N = 16) or were members of a recreation club for older adults for at least 6 months (non-institutionalized group: N = 16). In each group, half of the sample was randomly assigned to HRV biofeedback training and the other half to control training.

The exclusion criteria were as follows: a history of serious psychiatric, neurological, respiratory, or cardiovascular diseases (self-reported in the health questionnaire); cognitive impairments (score above the cut-off point in the Mini-Mental State Examination)^30^; severe waking difficulties; smoking; and regular use of medications acting upon the central nervous system. The study was conducted in accordance with the Declaration of Helsinki. The protocol was approved by the Research Ethics Committee of the Federal University of Ouro Preto (CAAE: 85839018.9.0000.5150) and all volunteers signed the informed consent form. All data were collected before the COVID-19 pandemic.

### Aerobic conditioning assessment

The sub-maximal aerobic capacity test, included in the Rikli and Jones battery of tests^31^, was used. It involves measuring the longest distance covered in a 6-min walk. The Physical Activity Readiness questionnaire^32^ and the Cardiovascular Risk Stratification (HHQ) questionnaire^33^ were used to select the individuals able to safely perform the aerobic test.

### Anthropometric assessment

The following variables were assessed: height (measured with a portable vertical stadiometer, model Balmak—EST-223, with a range of 0–2.1 m and precision of 1 mm), body mass (measured using an Omron digital scale, model Hn 289, with a capacity of 150 kg and precision of 100 g^34^), body mass index (BMI) (measured using the data of body mass (kg) and height (m) and the equation: weight/height^2^^34^), and body fat percentage determined using the Durnin and Womersley equation^35^, which uses four skinfolds in women (Db = 1.1339 – 0.0648 × log10 (subscapular skinfold + tricipital skinfold + suprailiac skinfold + bicipital skin fold)) and in men, it is calculated as Db = 1.1765 – 0.0744 × log10 (subscapular skinfold + tricipital skinfold + suprailiac skinfold + bicipital skinfold)). Subsequently, skinfold was converted into body fat percentage (%F) using the Siri equation (%F = [(4.95/SF) – 4.50] × 100))^36^.

### Emotional assessment

The social touch scale^37^ is composed of 28 items and was used to assess the frequency in which the participant performed or received social touches over the last 12 months. Scores ranged from 14 to 98 points in the scales of touching and being touched, whereas total touch scores vary between 28 and 196 points. There is no cut-off point in the scale. The higher the obtained score, the greater the individual’s ability to perform or receive social contact through touch.

The revised UCLA Loneliness Scale^38^ is a self-evaluation instrument with 20 items about feelings or actions related to loneliness. The higher the score, the stronger the feeling of perceived loneliness.

The Geriatric Depression Scale^39^ is composed of 15 items and assesses symptoms of depression in the older adults. The sum of the scores of the answers from each participant may vary between 0 and 15 points, and a score of 5 or higher indicates the likelihood of depression.

### HRV assessment

The electrocardiogram (ECG) recording was performed at rest, for 5 min, using the Nexus® 10 device, version 1.2, at three different moments: before and after training (which lasted 4.5 weeks), and on the follow-up (after 4.5 weeks). The unit of time was 1 ms, and the RR interval samples were collected at a sampling frequency of 256 Hz. The ECG electrodes were placed according to the Nexus-10® instructions, i.e., on the chest below the nipples and the clavicle because these points generate fewer artefacts. To prepare the skin for electrode placement, the pertinent areas of the skin were cleaned with ethanol.

Following data acquisition, HRV was extracted using the Kubios HRV Analysis Software (MATLAB, version 2.0 beta, Kuopio, Finland). In Kubios, the series was examined manually and inspected for artefacts. The following HRV components were analyzed: the standard deviation of all normal RR intervals recorded in a time interval (SDNN), which represents overall HRV activity and root mean square of the successive differences between RR intervals (RMSSD), percentage of the successive differences between the RR intervals that are greater than 50 ms (pNN50), standard deviation of instantaneous RR intervals (SD1) and high frequency (HF), which represents the predominance of parasympathetic activation^4^.

### Training

#### HRV biofeedback training

The Nexus-10® hardware (Mind Media BV) and the BioTrace® software were used to conduct the HRV biofeedback sessions, in addition to recording the RR intervals during training and at rest. During training, the computer screen showed the respiratory rate and heart rate graphs in real-time and the participant was instructed to try to increase the synchronization between heart and respiratory waves trying to increase the amplitude and decrease the frequency of the both waves. For that purpose, the software calculated the resonance frequency in real-time based on the calculation of Pearson’s correlation between heart rate and respiratory rate. The values of resonance frequency ranged from “1” (positive correlation between heart rate and respiratory rate) to “-1” (negative correlation between heart rate and respiratory rate). During the HRV biofeedback session, the participants were instructed to try to increase the resonance frequency. For that purpose, the reference value should be as close as possible to “1”. This training was adapted from the protocol proposed by Lehrer, Vaschillo and Vaschillo^1^ and consisted of 14 sessions of 15 min each, performed three times a week for a total of 4.5 weeks.

#### Control training

Three blocks of 50 images with neutral valence obtained from the catalogue of the International Affective Picture System^40^ were constructed. Within each block, the time of exposure of the photograph varied from 3 to 7 s, and then a blank screen was shown for 2–6 s; this variation in exposure time aimed to minimize habituation to the stimulus. The means of the three blocks did not differ significantly regarding valence (MV) and activation (MA) (Block 1: MV = 5.02; MA = 3.62, Block 2: MV = 5.02; MA = 3.40, Block 3: MV = 5.13; MA = 3.82) and were presented sequentially throughout the control training sessions (e.g., session 1 block 1, session 2 block 2, session 3 block 3, session 4 block 1, and so forth) so that the participants’ attention was engaged on the control task. The structure of this training was the same as that of the experimental training, consisting of 14 sessions of 15 min each, performed three times a week for 4.5 weeks.

An assessment was performed at the end of the training (biofeedback and control).

### Follow-up

**Figure 4:**
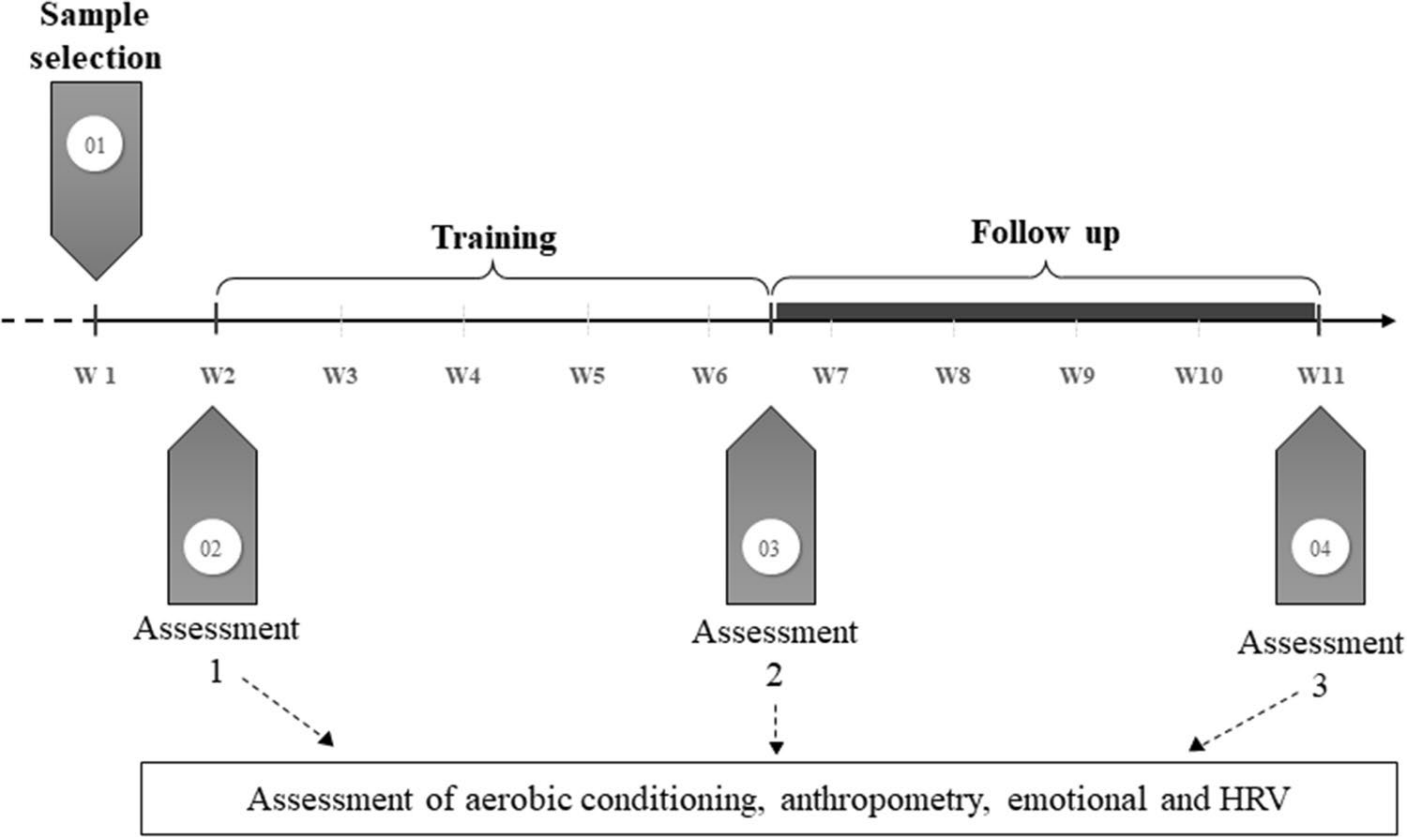
Experimental design.

A period of 4.5 weeks elapsed between the last training session and the re-assessment of the participants. The steps of the experimental design, from sample selection to the last assessment (follow-up assessment) are summarized in Fig. 4.

### Statistical analysis

The datasets used during the current study is available from the corresponding author on reasonable request. Initially, the normality of the raw data was tested using the Kolmogorov Smirnov test. Based on this, the normal (parametric) variables were described using the mean and standard deviation and the non-normal (non-parametric) variables were described using the median and the 25th and 75th percentiles. Subsequently, the t-test (normal variables) and the Mann–Whitney test (non-normal variables) were used for each collected variable (assessment 1) to determine the differences between the institutionalized group and the non-institutionalized group before starting any intervention.

To evaluate the differences between the collected variables before the start of the training (assessment 1), after the 14 training sessions (assessment 2), and after 4.5 weeks of follow-up (assessment 3) in the biofeedback and control groups within the non-institutionalized sample and then within the institutionalized sample, mixed-design repeated-measures ANOVA were performed for each parameter (anthropometric data, aerobic conditioning, emotional scores, and HRV components) using the factors time (assessments 1, 2, and 3) as the within variable and group (control and biofeedback) as the between variable. Subsequently, partial eta-squared was calculated to inform the effect size of all comparisons and Fisher’s post-hoc test was performed for the variables for which significant effects were observed. The SDNN, PNN50 and HF were normalized using logarithmic to use in the analyses.

The level of significance was set at 0.05 in all tests. Statistica 7.0 (StatSoft, Inc.) software was used in the analyses.

## Data Availability

The datasets used during the current study is available from the corresponding author on reasonable request.
